# Standardization of Beef, Pork, Chicken, and Soy Protein Extracts for Patch Testing and Their Accuracy in Diagnosing Adverse Food Reactions in Dogs with Chronic Pruritus

**DOI:** 10.3390/vetsci12040383

**Published:** 2025-04-18

**Authors:** Raniere Gaertner, Vanessa Cunningham Gmyterco, Júlia Só Severo, Camilla Alcalá, Maicon Roberto Paulo, Ruan Daros, Marconi Rodrigues de Farias

**Affiliations:** 1 Postgraduate Program in Animal Science, School of Medicine and Life Sciences, Pontifícia Universidade Católica do Paraná, 1155 Imaculada Conceição Street, Curitiba 80215-901, Brazil; raniere.gaertner@uniavan.edu.br (R.G.); vanessagmyterco@gmail.com (V.C.G.); juliasosevero@yahoo.com.br (J.S.S.); camilla_alcala@hotmail.com (C.A.); maiconpaulo@hotmail.com (M.R.P.); 2 EthoLab—Applied Ethology and Animal Welfare Lab, Graduate Program in Animal Science, School of Medicine and Life Sciences, Pontifícia Universidade Católica do Paraná, Curitiba 80215-901, Brazil; r.daros@pucpr.br

**Keywords:** cutaneous adverse food reactions, atopic dermatitis, food patch tests

## Abstract

(1): Background: This study aimed to evaluate the concentrations of four proteins for allergic patch testing (APT) in dogs, assessing sensitivity (SE), specificity (SP), negative predictive value (NPV), positive predictive value (PPV), reactions to adhesives/containers, and the safety of APT with food proteins in dogs. (2) Methods: For evaluation, 43 dogs were screened and divided into two groups: Group 1 consisted of 20 healthy dogs, and Group 2 included 23 dogs with canine atopic dermatitis (AD). Group 1 underwent allergic patch testing (APT) with beef, pork, chicken, and soy proteins at four different concentrations (100 mg, 250 mg, 500 mg, 1000 mg/0.2 mL). Of the 23 dogs included in Group 2, four did not undergo the elimination diet and were excluded, leaving 17 dogs in the study. They underwent an elimination diet (ED) and were evaluated using the pruritus visual analog scale (pVAS) and lesion scores (CADESI-4) before and after the ED (days 0 and 45). After the ED, Group 2 was subjected to APT (using the same proteins and concentrations as Group 1) and an oral provocation test (OPT) with the proteins used in the APT. The results of the OPT were used to assess the accuracy of the APT. (3) Results: In Group 1, one dog reacted to the APT. In Group 2, after 45 days of ED, of the 17 dogs included, 13 showed a reduction in pVAS and CADESI-4 scores (*p* < 0.05) and nine an improvement considered good to excellent. Of these, two showed irritant contact reactions to the APT chambers and were excluded, leaving 11 dogs that were reactive to APT, and the OPT increased pruritus (*p* < 0.05). Accuracy: Beef and chicken proteins at concentrations of 500 and 1000 mg/0.2 mL, and soy protein at 1000 mg/0.2 mL, achieved 100% SE, SP, PPV, and NPV. Pork protein at 1000 mg/0.2 mL achieved 100% SE, 83% SP, 83% PPV, and 100% NPV. (4) Conclusions: APT with beef and chicken proteins at 500 mg and 1000 mg/0.2 mL and soy protein at 1000 mg/0.2 mL, based on the results of this study, can be recommended for diagnosing adverse food reactions in dogs with AD.

## Simple Summary

This study evaluated different concentrations of beef, pork, chicken, and soy protein extracts for an atopic patch test in dogs. Twenty-three dogs with chronic pruritus were subjected to an elimination diet and evaluated on days 0 and 45 using the following lesion scores: Canine Atopic Dermatitis Extent and Severity Index (CADESI-4) and a pruritus visual analog scale (pVAS). They were then subjected to the atopic patch test with proteins at concentrations of 100, 200, 500, and 1000 mg/0.2 mL, followed by an oral provocation test. Beef, chicken, and soy proteins achieved 100% sensitivity, specificity, positive predictive value, and negative predictive value when used in higher concentrations. It was concluded that the atopic patch test at specific concentrations can be useful in diagnosing chronic pruritus associated with adverse reactions to beef, chicken, and soy proteins, as well as guiding the oral provocation test.

## 1. Introduction

Chronic and non-seasonal pruritus in dogs can be associated with adverse food reactions (AFRs) in approximately 30 to 76% of dogs with atopic dermatitis (AD) [1,2,3,4] and may involve immediate hypersensitivity responses mediated by IgE or delayed cell-mediated responses to various food components [2,5,6,7,8,9].

Currently, the “gold standard” for diagnosing adverse food reactions (AFRs) in dogs with chronic pruritus consists of implementing an elimination diet (ED) followed by an oral provocation test (OPT) [1,2,9,10,11,12].

However, since the elimination diet (ED) is labor-intensive, lengthy, challenging to implement, and costly, it is often associated with dropouts, making it necessary to seek diagnostic methods that are accessible, quick, and have good accuracy rates [13].

Delayed reactions mediated by lymphocytes are more common (84%) than IgE-mediated reactions in dogs with food allergies (FA), which makes the lymphocyte proliferation test exhibit high sensitivity, specificity, and positive predictive value in the diagnosis of food allergies in dogs [14].

Additionally, the allergic patch test (APT) with food has been used in medicine as an auxiliary examination for diagnosing food allergies (FA), particularly in individuals with atopic dermatitis (AD) [15]. However, the lack of standardization and low specificity of APT has led to it not being widely recommended in clinical practice [16], though it is considered an auxiliary tool for assessing the relationship between dermatological symptoms and FA in children with AD [17].

In dogs with chronic pruritus, often associated with atopic dermatitis, when APT is performed alone or in combination with the skin prick test, it shows high negative predictive values (NPVs), and its results have been used to select components for restrictive diets [11].

Thus, standardizing the concentrations of food extracts used in APT and its interpretation is necessary to improve its accuracy and enable its use in clinical practice for dogs with chronic pruritus [15,18].

This study aims to determine the appropriate concentrations of chicken, beef, pork, and soy protein extracts to be used in the allergic patch test with food (atopy patch test) for dogs with chronic pruritus associated with AFRs and to evaluate the sensitivity (SE), specificity (SP), negative predictive value (NPV), positive predictive value (PPV), safety, and harmlessness of the test.

## 2. Materials and Methods

The present study was a cross-sectional, controlled, and non-randomized study. It was approved by the Ethics Committee for Animal Use in Research (CEUA-PUCPR) under registration number 02256.

### 2.1. Experimental Group

To evaluate the irritant threshold concentrations of food extracts, dogs were screened regardless of breed and sex, with an age of over one year and a weight of over three kilograms. These dogs were divided into two study groups. Group 1 consisted of 20 healthy dogs, with no previous history of dermatological or systemic diseases. Group 2 included 23 dogs with a history of chronic, primary, perennial pruritus, ranging from moderate to severe, localized in interdigital, axillary, abdominal, and inguinal regions. These dogs had a history of recurrent otitis, and some also had perioral and perianal pruritus. The presence of pruritic dermatopathies of infectious or parasitic origin, or flea allergy dermatitis, was ruled out through complementary diagnostic tests, such as parasitological skin scraping and skin cytology. Only dogs that met at least six of the eight clinical criteria established by Favrot et al. (2010) [19] were included in Group 2.

All dogs received monthly endoparasite control with praziquantel, febantel, and pyrantel (Duprantel plus^®^, Duprat; Descalvado, Brazil), without palatability enhancers, and ectoparasite control with a topical fipronil-based product (Effipro^®^ “spot on”, Virbac; Carros, France) every 14 days, along with an up-to-date vaccination protocol. The dogs were screened at the Veterinary Clinic of the School of Veterinary Medicine at PUCPR (Paraná, Brazil) and a Veterinary Dermatology Clinic at Pet Center (Santa Catarina, Brazil). Dogs were excluded if they had used systemic glucocorticoids within four weeks, or topical glucocorticoids and antihistamines within two weeks prior [11]. Exclusion criteria also included chronic atopic dermatitis with lichenification in the APT application area, discomfort, pruritus, or behavioral changes during the APT, or if they removed the tape or food extracts or if owners did not follow the protocol.

### 2.2. Experimental Protocol

#### 2.2.1. Preparation of the Experimental Groups and Elimination Diet

The owners of the dogs in Group 2 provided information regarding the degree of pruritus by marking a value on the pruritus visual analogue scale (pVAS) [20], where 0 indicates no pruritus and 10 indicates intense pruritus. Following this, the dogs underwent a clinical examination and lesion evaluation according to the Canine Atopic Dermatitis Extent and Severity Index (CADESI-4 scale) [21]. The dogs in Group 2, starting on day 0, were subjected to an elimination diet (ED) consisting of 190 mL of lentils and half a cup (190 mL) of sweet potato per five kilograms of body weight, administered daily [22]. Initial control of pruritus in the dogs was achieved with oclacitinib maleate (Apoquel) at a dose of 0.5 mg/kg orally once daily (SID) for 15 days, alongside the elimination diet. After 15 days, the medication was discontinued, and pruritus and lesions were reassessed on day 30. If improvement was observed, the diet was extended until day 45. If there was a worsening of pruritus on day 30 after discontinuation of treatment, the dogs were medicated again with oclacitinib maleate for another seven days. This treatment was then discontinued on day 37, and the dogs were re-evaluated at 60 days. Upon further improvement, the elimination diet was discontinued, and the oral provocation test was restarted. Clinical improvement was assessed based on pre-established lesion and pruritus scores on days 0 and 45 or 60 of the ED. The response to the pVAS evaluation was categorized as follows: (a) no improvement, (b) reduction up to 50%: moderate, (c) reduction from 50.1% to 80%: good, (d) reduction from 80.1% to 90%: very good, and (e) reduction of pruritus above 90%: excellent [23].

#### 2.2.2. Atopic Patch Test

Dogs from both groups were subjected to the atopic patch test (APT) with extracts made from raw beef, pork, chicken, and cooked soy. The dogs in Group 1 were tested starting on day 0, while the dogs in Group 2 were tested after the elimination diet (ED).

#### 2.2.3. Preparation of the Food Extracts

The extracts for the APT were prepared from raw beef, pork, chicken, and cooked soy, which were processed in a Philips Walita RI7636/750W food processor (Philips Electronics Nederland B.V., Eindhoven, Netherlands), homogenized in semi-solid petroleum jelly at four different concentrations: a-100 mg/0.2 mL, b-250 mg/0.2 mL, c-500 mg/0.2 mL, d-1000 mg/0.2 mL. They were then stored in 20 mL plastic syringes (10 g of extract in each syringe). The meats used for the preparation of allergic extracts were purchased from a butcher shop and originated from slaughterhouses under federal inspection. The cuts used were chuck steak, pork leg, and chicken breast. Microbiological tests were not performed on the meats used; however, the processing of the allergic extracts was carried out on the same day as the purchase of the animal proteins and was always kept frozen until use.

#### 2.2.4. Preparation of the Containers and Patch Test with Food Extracts

For the preparation of the APT with food extracts, container chambers with 10 wells, each 8 mm in diameter, were used, adhered to a hypoallergenic tape (AllergoChamber^®^, neoflex, Sertãozinho-SP, Brazil).

For the execution of the APT, dogs from both groups were subjected to clipping with a #40 blade on the right hemithorax, an area approximately 20 cm × 15 cm, 48 h before the test, to minimize irritating reactions [18].

On the day of the test, the chambers were prepared and fixed onto the skin after a light cleaning with saline solution. The AllergoChamber tapes were fixed in a dorsoventral direction, placed side by side to guide the reading of possible reactions. The first row, in the dorsoventral direction, was composed of beef extracts; the second row contained pork extracts; the third row contained chicken extracts; and the fourth row contained soy extracts. Each row consisted of four wells, which were filled with extracts of increasing concentrations of each food tested. The last chamber of the first column was filled with petroleum jelly (negative control).

After fixation, the AllergoChambers were covered with 12 cm crepe bandages and 10 cm adhesive tape over the bandages. The dogs were then dressed in specific surgical clothing [11].

#### 2.2.5. Interpretation of the Patch Test with Food Extracts

The AllergoChamber tapes were removed after 48 h, and 15 min later, the reactions were interpreted based on the criteria established by Bethlehem et al. (2012) [18] (Table 1).

#### 2.2.6. Oral Provocation Test (OPT) in Group 2 Animals

To rule out an irritative effect of the food extract, reactions to microbial toxins present in the extract, or contact dermatitis from the containment chambers used in the APT, after evaluating the response to the elimination diet (ED) and the APT, the adverse skin reaction to food in the dogs of Group 2 was only confirmed after undergoing the oral provocation test (OPT) with each tested protein, regardless of their APT results. They were monitored until clinical signs reappeared or, if no recurrence occurred, for a maximum of 14 days.

The cooked protein used in the OPT was incorporated into the elimination diet (ED) at a daily dose of approximately 10 to 20 g per meal.

If clinical signs recurred after the oral provocation test (OPT), the offending food was discontinued, and the patient was treated with oclacitinib (Apoquel^®^, Zoetis; São Paulo, Brazil) at a dose of 0.5 mg/kg orally every 12 h for up to three days. The elimination diet (ED) was then resumed without the offending protein. A new OPT was performed only one week after discontinuing treatment, once pruritus was controlled and the patient showed clinical improvement.

#### 2.2.7. Statistical Analysis

Demographic data, including breed and sex, are presented as absolute values and percentages. Age data are presented as mean and standard deviation.

Data related to the APT are described as the percentage of reactions to food extracts.

Evaluations of the pruritus (pVAS) and lesional (CADESI-4) scales are presented as mean and standard deviation.

Analysis of the scores of these scales over time was performed using repeated measures analysis of variance, with a 95% confidence interval and *p* < 0.05, using the paired *t*-test.

Diagnostic performance (sensitivity, specificity, positive and negative predictive value) of the APT, with the four proteins at the four concentrations and different reactions considered positive, was calculated using contingency tables.

Analyses were conducted using the R statistical software (R Core Team v 4.1.2, 2019).

## 3. Results

### 3.1. Demographic and Epidemiological Data

There was no sample loss in Group 1. Of these, nine (45%) were mixed breed and eleven (55%) were of a specific breed; the mean age was 7.95 years with a standard deviation of 3.65 years, consisting of thirteen (65%) males and seven (35%) females.

In Group 2, of the 23 dogs that started the study, six owners discontinued the elimination diet (ED) and dropped out. Of the seventeen dogs that completed the ED, fifteen were of a specific breed (88.2%) and two were mixed breed (11.8%), with nine (52.9%) males and eight (47.1%) females. The mean age was 7.29 years with a standard deviation of 2.58 years. The demographic data of the dogs included at the beginning of the experimental protocol can be observed in Table 2.

### 3.2. Reactions Observed in Group 1 Dogs

In the evaluation of reactions to the APT with the four tested food extracts at various concentrations, only one of the 20 healthy dogs (5%) exhibited reactions identified as mild erythema (1+) to beef protein at concentrations of 500 mg/0.2 mL and 1000 mg/0.2 mL.

### 3.3. Response to Elimination Diet (ED) in Group 2 Dogs

Of the 17 dogs that continued with the elimination diet (ED), four (23.52%) did not show significant improvement.

The remaining 13 dogs (76.47%) in Group 2 demonstrated percentage improvement in pruritus and lesion scores.

The mean pVAS score for these 13 dogs on day 0 was 8.61, which decreased to 2.96 by day 45, representing a 66% reduction in pruritus (*p* = 2.651 × 10^−10^) (Table 3). Individually, four dogs (30.8%) showed a reduction of up to 50%, classified as “regular”; six dogs (46.1%) experienced a reduction between 50.1% and 80%, classified as “good”; two dogs (15.4%) had a reduction between 80.1% and 90%, classified as “very good”; and one dog (7.7%) achieved a reduction between 90.1% and 100%, classified as “excellent”.

The mean CADESI-4 score for these 13 dogs on day 0 of the diet was 18.54, which decreased to 8.46 by day 45, reflecting a 54.4% reduction in lesion scores (*p* =2.90037 × 10^−9^).

### 3.4. Reactions to APT in Group 2 Dogs After ED

The four dogs that did not show significant improvement with the elimination diet (ED) were subjected to the APT with food extracts. After 48 h, all tests were negative for the proteins and concentrations evaluated.

Of the 13 dogs that improved with the ED and underwent the APT, two exhibited severe reactions during the test, showing restlessness, discomfort, and intense pruritus. These dogs were removed from the experimental protocol and appropriately treated, therefore, a total of 11 dogs from Group 2 completed the experimental protocol.

Among the 11 dogs that remained in the experimental protocol, eight (72.72%) reacted to chicken and soy, and six (54.54%) to beef and pork.

Analyzing the intensity of the positive reactions, out of a total of seventy-nine reactions, fifty-five (69.62%) were mild erythema, seventeen (21.52%) moderate erythema, four (5.06%) intense erythema, one (1.26%) intense erythema associated with micropapules and plaques, and two (2.53%) intense erythema with microvesiculations and erosions.

Of the eleven animals that completed the experimental protocol, eight (72.7%) reacted to more than one tested protein. Specifically, three (27.3%) reacted to all four proteins, three (27.3%) reacted to three proteins, two (18.2%) reacted to two proteins, and three (27.3%) reacted to only one tested protein.

The reaction scores for each concentration of the tested food extracts are shown in Table 4.

### 3.5. Reactions to the Tape and Chambers Used

Of the seventeen dogs that underwent the APT, six (35.29%) exhibited irritant contact reactions to the adhesive of the AllergoChambers^®^, with erythema observed in the spaces between the container chambers and other adhesive contact points on the previously shaved skin. In three (50%) of these six dogs, reactions to the plastic containers were noted, characterized by erythema associated with annular or arciform edema.

### 3.6. Evaluation of the OPT in Patients from Group 2

The eleven dogs that improved with the ED and reacted to the APT exhibited recurrence of clinical signs after exposure to the foods they reacted to (*p* < 0.05, where *p* = 4.12861 × 10^−6^), except for one dog (9.09%) that reacted to pork meat in the APT, and the OPT with this protein was performed and extended for 14 days, without recurrence of clinical signs.

The values of the pVAS on day 0 and on the 45th day of the ED, as well as after the OPTs, including mean, standard deviation, and minimum and maximum values, are shown in Table 5.

There was no recurrence of clinical signs after the OPT in any of the dogs when they were challenged for 14 days with the foods that tested negative in the contact test (Table 6).

### 3.7. Evaluation of the Accuracy Indices of the APT

In the 11 dogs where the ATP with food was positive and symptoms recurred with the oral provocation test, the accuracy indices of the test were evaluated.

The assessment of sensitivity (SE), specificity (SP), positive predictive value (PPV), and negative predictive value (NPV), considering the reaction scores and each concentration of the tested foods, is presented in Table 7.

## 4. Discussion

Unfortunately, adverse reactions to the atopic patch test in two patients, non-adherence to the elimination diet by six owners, and the lack of response to the elimination diet in four patients reduced the number of dogs with atopic dermatitis in Group 2 from 23 to 11. This highlights the difficulty in conducting elimination diets and OPT, as well as in reaching a diagnosis of adverse food reactions.

Recent studies in dogs with chronic pruritus and atopic dermatitis (AD) emphasize the role of food allergens in AD’s pathogenesis. Research shows that 67% of dogs with chronic pruritus are co-sensitized to environmental and food allergens, with up to 76% showing partial improvement on an elimination diet (ED) [2,24,25]. The prevalence of AFRs in dogs with AD ranges from 30 to 76% in different articles [1,2,3,4], and studies suggest that EDs formulated based on patch test results or their combination with prick tests appear to yield better outcomes than EDs based on dietary history [2].

In this study, 52,94% of dogs with chronic pruritus had good to excellent improvement with the exclusion diet, and all meeting AD diagnostic criteria. Additionally, 72.7% of dogs that completed the protocol were sensitized to more than one tested dietary protein. This suggests that dogs with AD may become sensitized to regularly exposed food allergens, and the institution of maintenance diets that do not contain allergens that these dogs react to may be necessary to control the disease [2,26].

The foods most associated with hypersensitivity in this study were chicken and soy, consistent with another study conducted in Brazil [2]. Chicken is a protein source frequently used in commercial pet foods, while soy is a common substitute for animal protein in these diets, which may contribute to higher sensitization rates to these proteins.

A single positive result in APT, including high concentrations of extracts, among 20 healthy dogs, indicates good specificity of the test, suggesting that positive reactions are more likely to occur in dogs with AD, possibly due to dysfunctions in the skin’s physical barrier and hyperreactivity and proliferation of lymphocytic subpopulations [2,27,28,29], and the high positive predictive values (PPVs) and specificity (SP) observed in Group 2 dogs to beef, chicken, and soy extracts likely represent specific immunoallergic and inflammatory reactions in dogs with AD, rather than irritant contact dermatitis [15].

Lower concentrations, such as 100 mg and 250 mg/0.2 mL with the tested proteins, showed great variability in accuracy values and, overall, demonstrated lower sensitivity, specificity, positive predictive value, and negative predictive value compared to higher concentrations. These lower concentrations are not suitable for use in APT in dogs with chronic pruritus.

Mild erythema was the most observed positive reaction, accounting for 69.62% of cases, followed by moderate erythema at 21.52%.

The relationship between higher reaction scores and concentrations varied depending on the protein tested, which may indicate that certain food allergens require higher concentrations to achieve better specificity and positive predictive value [2,11].

Thus, considering mild erythema as a positive reaction, it was found that beef and chicken proteins at concentrations of 500 mg and 1000 mg/0.2 mL, and soy protein at a concentration of 1000 mg/0.2 mL, appear to be suitable for use in allergy patch tests, achieving excellent values for SE, SP, PPV, and NPV.

In contrast, pork protein achieved the highest SE, SP, PPV, and NPV values at a concentration of 1000 mg/0.2 mL, with SE and NPV reaching 100%. However, SP and PPV were both at 83%, indicating that at this concentration, pork protein may induce false-positive results [2], making positive results useful only for its selection in elimination diets (ED).

Higher concentrations of the tested proteins resulted in a greater number of reactive animals and reactions with higher scores and the best accuracy values was 1000 mg/0.2 mL for all four tested proteins. However, as concentrations of 500 mg/0.2mL for beef and chicken have already produced significant and easily interpreted responses, perhaps these concentrations are already sufficient for patch test performance with these proteins. It is important to highlight that the 1000 mg/0.2 mL concentrations induced more pronounced and easily interpretable reactions, without causing irritation in the patients from Group 1.

Regarding the safety of the test, healthy dogs did not exhibit any discomfort during the procedure. Reactions to the adhesives and containers were observed in 35.29% of dogs with AD, and in 11.8% of cases, the test had to be removed due to exacerbated reactions and discomfort. Dogs with AD are more susceptible to irritant contact reactions to the materials used in the APT, which can lead to premature removal, prevent result interpretation, or increase the likelihood of false positives.

Future studies evaluating the irritative threshold and the optimal concentrations of other proteins are needed to improve the atopic patch test. Additionally, studies should consider using containment patches without food allergen extracts to more accurately evaluate their potential to induce irritant contact dermatitis in dogs with atopic dermatitis (AD). This approach will allow for a more precise determination of whether the observed reactions are exclusively related to the containment patches.

Exacerbated reactions to APT could limit its use [2,15]. Therefore, after the test, the reactive nature of atopic skin requires outpatient observation for 20 to 30 min to monitor tolerance [30]. Owners should also be advised to report any local itching or discomfort immediately.

In conclusion, based on the accuracy values obtained in this study, beef and chicken proteins at 500 mg and 1000 mg/0.2 mL and soy protein at 1000 mg/0.2 mL can be recommended for diagnosing adverse food reactions in dogs with AD, helping with food selection for exclusion diets and for challenges during the oral provocation test.

## Figures and Tables

**Table 1:** Scoring of lesions in patch tests in dogs.

0 = no visible reaction or irritation;
1+ = mild erythema;
2+ = moderate erythema;
3+ = intense erythema;
4+ = erythema and plaque;
5+ = erythema with vesiculation and/or pustules, or more intense lesions

Modified from Bethlehem et al., 2012 [18].

**Table 2:** Demographic data of the dogs included at the beginning of the experimental protocol.

Group 1		Group 2	
n (20)		n (23)	
Breed		Breed	
Maltese Terrier	1	Golden Retriever	2
Poodle	6	Border Collie	1
Schnauzer	1	French Bulldog	1
Shih Tzu	2	English Bulldog	1
Yorkshire Terrier	1	Maltese Terrier	1
Mixed breed	9	Poodle	2
		Pug	1
		Shih Tzu	5
		Yorkshire Terrier	4
		SRD	5
Gender		Gender	
Male (neutered)	13	Male (neutered)	10
Female (neutered)	7	Male (intact)	1
		Female (neutered)	12
Age (mean)	7.95	Age (mean)	6.14

**Table 3:** Means, percentage reduction, and standard deviation compared to day 0 (D0) of Canine Atopic Dermatitis Extent and Severity Index (CADESI-4) and pruritus visual analogue scale (pVAS) scores of Group 2 dogs (N = 13) subjected to the elimination diet (ED).

	pVAS			CADESI-4		
Time	pVAS (Mean)	Percentage Reduction (Compared to T0)	Pvas (SD)	CADESI (Mean)	Percentage Reduction (Compared to T0)	CADESI (SD)
D0	8.61	-	1.040	18.54	-	2.818
D45	2.96	66%	1.473	8.46	54.4%	1.337

**Table 4:** Reaction scores for each concentration (mg/0.2 mL) of the tested food extracts (beef, pork, chicken, and soy) in the 11 evaluated dogs that continued in the study.

**Protein**	**Beef**				**Pork**			
	100	250	500	1000	100	250	500	1000
**Reaction**								
**1+**	1	2	2	1		1	3	2
**2+**		1	2	3			2	4
**3+**		1	1	1				
**4+**		1						
**5+**			1	1				
**Total**	1	5	6	6		1	5	6
**Protein**	**Chicken**				**Soy**			
	100	250	500	1000	100	250	500	1000
**Reaction**								
**1+**	4	7	6	5	1	6	7	7
**2+**			2	2				1
**3+**				1				
**4+**								
**5+**								
**Total**	4	7	8	8	1	6	7	8

**Table 5:** Mean pruritus visual analog scale (pVAS) values on day 0 and day 45 of the ED, and mean values of positive OPTs (N:11).

Diet Day	Mean	SD	Minimum	Maximum
**D0**	8.681	1.113	7	10
**D45**	2.863	1.568	0	5
**OPT+**	6.918	0.791	5,5	8

**Table 6:** Number of animals and the relationship between the tested protein (regardless of concentration), the result of the atopic patch test with food, and the oral provocation test in the 11 dogs from Group 2 that completed the study.

Protein	Positive APT	Positive OPT	Negative APT	Negative OPT
**Chicken**	8	8	3	3
**Soy**	8	8	3	3
**Beef**	6	6	5	5
**Pork**	6	5	5	6

**Table 7:** Accuracy values for the four concentrations of beef, pork, chicken, and soy, considering as positive reactions sequentially >1+, >2+, >3+, >4+, >5+.

Beef Protein Positive ≥ 1+	SE%	SP%	PPV%	NPV%
**100 mg/0.2 mL**	17 (0–64)	100 (48–100)	100 (3–100)	50 (19–81)
**250 mg/0.2 Ml**	83 (36–100)	100 (48–100)	100 (48–100)	83 (36–100)
**500 mg/0.2 mL**	100 (54–100)	100 (48–100)	100 (54–100)	100 (48–100)
**1000 mg/0.2 mL**	100 (54–100)	100 (48–100)	100 (54–100)	100 (48–100)
**Beef protein positive ≥ 2+**				
**100 mg/0.2 mL**	100 (54–100)	0 (0–52)	55 (23–83)	NA * (0–100)
**250 mg/0.2 mL**	50 (12–88)	100 (48–100)	100 (29–100)	62 (24–91)
**500 mg/0.2 mL**	67 (22–96)	100 (48–100)	100 (40–100)	71 (29–96)
**1000 mg/0.2 mL**	83 (36–100)	100 (48–100)	100 (48–100)	83 (36–100)
**Beef protein positive ≥ 3+**				
**100 mg/0.2 mL**	100 (54–100)	0 (0–52)	55 (23–83)	NA * (0–100)
**250 mg/0.2 mL**	33 (4–78)	100 (48–100)	100 (16–100)	56 (21–86)
**500 mg/0.2 mL**	33 (4–78)	100 (48–100)	100 (16–100)	56 (21–86)
**1000 mg/0.2 mL**	33 (4–78)	100 (48–100)	100 (16–100)	56 (21–86)
**Beef protein positive ≥ 4+**				
**100 mg/0.2 mL**	100 (54–100)	0 (0–52)	55 (23–83)	NA * (0–100)
**250 mg/0.2 mL**	17 (0–64)	100 (48–100)	100 (3–100)	50 (19–81)
**500 mg/0.2 mL**	17 (0–64)	100 (48–100)	100 (3–100)	50 (19–81)
**1000 mg/0.2 mL**	17 (0–64)	100 (48–100)	100 (3–100)	50 (19–81)
**Beef protein positive ≥ 5+**				
**100 mg/0.2 mL**	100 (54–100)	0 (0–52)	55 (23–83)	NA * (0–100)
**250 mg/0.2 mL**	100 (54–100)	0 (0–52)	55 (23–83)	NA * (0–100)
**500 mg/0.2 mL**	17 (0–64)	100 (48–100)	100 (3–100)	50 (19–81)
**1000 mg/0.2 mL**	17 (0–64)	100 (48–100)	100 (3–100)	50 (19–81)
**Pork protein positive ≥ 1+**	**SE%**	**SP%**	**PPV%**	**NPV%**
**100 mg/0.2 mL**	100 (48–100)	0 (0–46)	45 (17–77)	NA * (0–100)
**250 mg/0.2 mL**	0 (0–52)	83 (36–100)	0 (0–97)	50 (19–81)
**500 mg/0.2 mL**	80 (28–99)	83 (36–100)	80 (28–99)	83 (36–100)
**1000 mg/0, 2mL**	100 (48–100)	83 (36–100)	83 (36–100)	100 (48–100)
**Pork protein positive ≥ 2+**				
**100 mg/0.2 mL**	100 (54–100)	0 (0–52)	55 (23–83)	NA * (0–100)
**250 mg/0.2 mL**	100 (54–100)	0 (0–52)	55 (23–83)	NA * (0–100)
**500 mg/0.2 mL**	40 (5–85)	100 (54–100)	100 (16–100)	67 (30–93)
**1000 mg/0.2 mL**	80 (28–99)	100 (54–100)	100 (40–100)	86 (42–100)
**Chicken protein positive ≥ 1+**	**SE%**	**SP%**	**PPV%**	**NPV%**
**100 mg/0.2 mL**	50 (16–84)	100 (29–100)	100 (40–100)	43 (10–82)
**250 mg/0.2 mL**	88 (47–100)	100 (29–100)	100 (59–100)	75 (19–99)
**500 mg/0.2 mL**	100 (63–100)	100 (29–100)	100 (63–100)	100 (29–100)
**1000 mg/0.2 mL**	100 (63–100)	100 (29–100)	100 (63–100)	100 (29–100)
**Chicken protein positive ≥ 2+**				
**100 mg/0.2 mL**	100 (63–100)	0 (0–71)	73 (39–94)	NA * (0–100)
**250 mg/0.2 mL**	100 (63–100)	0 (0–71)	73 (39–94)	NA * (0–100)
**500 mg/0.2 mL**	25 (3–65)	100 (29–100)	100 (16–100)	33 (7–70)
**1000 mg/0.2 mL**	38 (9–76)	100 (29–100)	100 (29–100)	38 (9–76)
**Chicken protein positive ≥ 3+**				
**100 mg/0.2 mL**	100 (63–100)	0 (0–71)	73 (39–94)	NA * (0–100)
**250 mg/0.2 mL**	100 (63–100)	0 (0–71)	73 (39–94)	NA * (0–100)
**500 mg/0.2 mL**	100 (63–100)	0 (0–71)	73 (39–94)	NA * (0–100)
**1000 mg/0.2 mL**	12 (0–53)	100 (29–100)	100 (3–100)	30 (7–65)
**Soy protein positive ≥ 1+**	**SE%**	**SP%**	**PPV%**	**NPV%**
**100 mg/0.2 mL**	12 (0–53)	100 (29–100)	100 (3–100)	30 (7–65)
**250 mg/0.2 mL**	75 (35–97)	100 (29–100)	100 (54–100)	60 (5–95)
**500 mg/0.2 mL**	88 (47–100)	100 (29–100)	100 (59–100)	75 (19–99)
**1000 mg/0.2 mL**	100 (63–100)	100 (29–100)	100 (63–100)	100 (29–100)
**Soy protein positive ≥ 2+**				
**100 mg/0.2 mL**	100 (63–100)	0 (0–71)	73 (39–94)	NA * (0–100)
**250 mg/0.2 mL**	100 (63–100)	0 (0–71)	73 (39–94)	NA * (0–100)
**500 mg/0.2 mL**	100 (63–100)	0 (0–71)	73 (39–94)	NA * (0–100)
**1000 mg/0.2 mL**	12 (0–53)	100 (29–100)	100 (3–100)	30 (7–65)

* Does not apply.

## Data Availability

Data are available upon request to raniere.gaertner@uniavan.edu.br.
